# Knowledge, attitude and practice of kangaroo mother care among mothers in the neonatal wards of a tertiary care center

**DOI:** 10.11604/pamj.2021.38.364.22833

**Published:** 2021-04-14

**Authors:** Olubukola Olawuyi, Beatrice Nkolika Ezenwa, Iretiola Bamikeolu Fajolu, Mercy Onwuama, Chinyere Veronica Ezeaka

**Affiliations:** 1 School of Post-Basic Nursing, Lagos University Teaching Hospital, Lagos, Nigeria,; 2 Department of Pediatrics, College of Medicine University of Lagos, Lagos, Nigeria,; 3 Department of Human Kinetics and Health Education, University of Lagos, Lagos, Nigeria

**Keywords:** Kangaroo mother care, preterm infant, mothers, low birth weight, Lagos

## Abstract

**Introduction:**

approximately 1 million children die each year due to complications of preterm birth with the major contributor to mortality being hypothermia. Kangaroo mother care (KMC) is an effective and low-cost technique which prevents neonate from hypothermia. The mother uses her body temperature to keep the infant warm thereby preventing demise from cold injury. Not much is known about the perception and practice of this simple and easy method of caring for preterm infants among post-natal mothers in Nigeria. This study aimed to determine the knowledge, attitude and practice of kangaroo mother care among mothers in the neonatal wards of a tertiary care center in Nigeria.

**Methods:**

this study was a hospital-based descriptive cross-sectional survey of sixty mothers selected from the Neonatal ward of the Lagos University Teaching Hospital (LUTH), Idi-Araba using convenient sampling technique. Data was collected with the use of a questionnaire and analyzed using descriptive statistics. Frequency and percentages were presented in tables and chi-square was used to test associations between categorical variables; p-value <0.05 was considered significant.

**Results:**

the findings revealed that 80% of respondents had heard of kangaroo mother care with 66.6% having good knowledge. Two-thirds (65%) of the respondents had a good attitude towards the use of KMC with 71.7% feeling happy when their baby is in kangaroo position. The knowledge of mothers significantly influenced their attitude and practice of KMC, p <0.05.

**Conclusion:**

the knowledge of KMC among mothers whose babies were admitted into the newborn wards of LUTH was high and they believe that KMC is helpful to their babies and were happy practicing it.

## Introduction

Over 15 million infants are born preterm every year and over 96% of them in developing countries [1]. Prematurity is the leading cause of death in under-5 children. It accounts for 60-90% of newborn deaths globally [2] with nearly 1 million deaths annually from the complications of prematurity [3]. In low-income settings, half of the babies born at or below 32 weeks die due to lack of feasible, affordable and effective low-cost care, such as provision of warmth, breastfeeding support, and basic care for infections and breathing difficulties [1]. While not all premature babies experience complications, generally, the earlier the gestation, the higher the risk of complications and the higher the risk of mortality. Some of the complications may be apparent at birth, such as respiratory distress and hypothermia and, require immediate attention. Hypothermia can lead to breathing problems, low blood sugar levels [4] and even death. Premature babies lose body heat rapidly and may require additional heat from external sources such as an incubator to maintain body temperature.

Kangaroo mother care (KMC) is another method of providing warmth for low birth weight (LBW) infants by placing the infant in skin-to-skin contact with the mother´s chest. The practice can provide thermal care, enhance nutrition and prevent sepsis in LBW infants [5]. KMC has been proposed as an alternative to conventional incubator care for LBW infants [6] that is effective and low-cost. The parent's stable body temperature helps to regulate the neonate's temperature more smoothly than an incubator and allows for readily accessible breastfeeding [7]. KMC was developed initially as a response to overcrowding, and insufficient resources in neonatal intensive care units (NICU) [8, 9]. Today, the World Health Organization has endorsed KMC as a standard of care for preterm, LBW infants [8]. A recent survey, documented 82% of NICUs in the United States to be using KMC [10].

Interestingly, the uptake of KMC in many resource-poor countries such as Nigeria has been sub-optimal and variable. In a study on the awareness and perception of KMC among mothers and the role of the healthcare providers in selected primary healthcare facilities in Calabar, Nigeria, Nsemo *et al*. noted that low awareness of KMC led to negative perception and poor practice of KMC [11]. Roba *et al*. in Eritrea, assessed the knowledge of postnatal mothers concerning KMC, and found that more than half (53%) of the postnatal mothers had poor knowledge [12]. Shruti *et al*. in India, reported that mothers with high knowledge had a more positive attitude towards KMC [13]. The inconsistency in the utilization of KMC despite its obvious benefits [14] should be concerning to all stakeholders. In Nigeria KMC was adopted and included in the integrated facility-based newborn care package from the late 1990s [15]. The present study aimed to assess the knowledge, attitude and practice of KMC among mothers in the neonatal wards of Lagos University Teaching Hospital, Lagos Nigeria. The findings from this study will contribute to data regarding the perception of post-natal mothers on KMC in Nigeria.

## Methods

**Study design and setting:** this was a hospital-based prospective and descriptive cross-sectional study carried out between September 1^st^ and October 31^st^, 2019 among post-natal mothers in the outborn neonatal wards of Lagos University Teaching Hospital (LUTH). LUTH receives neonatal referrals from other teaching hospitals, general hospitals, private hospitals, maternity homes as well as home deliveries in Lagos and environs. Mothers were an integral part of the care team for in-patients in the newborn wards and mothers with LBW infants were counselled and actively engaged to render KMC to their newborns once they were stable.

**Study population:** the study population comprised of all the 60 consecutive mothers of preterm infants admitted into the out born neonatal wards of LUTH during the two months´ study period. We included all the postnatal mothers with stable but low birth weight preterm infants whom the managing physicians had prescribed KMC for and who consented to participate in the study. We excluded mothers whose babies were not preterm or low birth weight, mothers who were sick and not available to provide KMC and mothers who were not willing to participate.

**Data collection:** eligible mothers were approached by one of the researchers and the study was explained. Thereafter, an informed consent to participate in the study was obtained if the mother was willing. A structured and pretested questionnaire was then administered to the mother to complete in two parts: before and after some KMC sessions. The questionnaires were structured into four sections: socio-demographic characteristics, knowledge, attitude and practice of kangaroo mother care. Mothers´ knowledge was sought for in definition, content and benefits of KMC. Closed-ended yes/no questions on their feelings and perceptions while on KMC were used to assess attitude while responses to practice questions such as techniques and duration of KMC were used to assess practices. Completed questionnaires were retrieved and checked for completeness and then entered into an excel spreadsheet.

**Data analysis:** Statistical Package for Social Sciences (SPSS) version 21.0 was used as the statistical tool for analysis after importing data from Excel. Descriptive statistics was employed and data were presented using frequency tables and percentages. Test of association between dependent variables such as knowledge, attitude and practice were analyzed against the independent variables using the Pearson chi-squared test. Findings were statistically significant if the p-value was <0.05.

**Ethical consideration:** this research was approved by the ethics and research committee of Lagos university teaching hospital, Lagos. Verbal and written consents were obtained from the mothers before administering the questionnaires.

## Results

Sixty mothers completed the study. Table 1 shows the socio-demographic characteristics of the participants. Majority of the respondents were of Yoruba ethnicity and were married. Most of the mothers were in the age bracket of 31 to 40 years. Forty-eight percent (80%) of the participants had heard of kangaroo mother care. Respondents´ source of information identified that 60% heard about kangaroo mother care for the first time in the hospital, 5% from social media, neighbors 5% and 10% from family members. Table 2 shows the respondents´ knowledge of KMC; 40 (66.6%) of the respondents correctly defined KMC as holding preterm LBW infants skin to skin to keep warm. Two-third (66.7%) of the respondents were knowledgeable about the benefits of the KMC.

**Table 1:** demographic data

Variables	Frequency (%) N = 60
**Age group (years)**	
16 - 20	3 (5)
21 - 30	18 (30)
31 - 40	36 (60)
Above 41	3 (5)
**Ethnic group**	
Yoruba	45 (75)
Igbo	15 (25)
Hausa	
Others	
**Occupation**	
Civil Servant	22 (36.7)
Trader	18 9 (30)
House wife	12 (20)
Student	8 (13.3)
**Marital status**	
Married	52 (86.6)
Single	
Divorced	4 (6.7)
Widow	4 (6.7)
**Qualification**	
Graduate	25 (42)
Secondary school certificate	5 (8)
Primary school	18 (30)
Not educated	12 (20)

**Table 2:** knowledge of mothers on the use of kangaroo mother care (KMC)

Variable	Frequency (%) N = 60
KMC is a way of carrying baby in front of Mother only	10 (16.7)
KMC is holding small babies skin-to-skin with caregiver to keep baby warm	40 (66.7)
KMC is putting hot water bottle in the cot to warm babies	10 (16.7)
KMC is keeping babies beside fire to warm them	-
**Which newborn requires kangaroo mother care**	
Pre-term	45 (75)
Fat baby	-
Post-term	-
Baby greater than 4kg	15 (25)
**Baby can breastfeed during KMC**	
Yes	35 (58.3)
No	25 (41.7)
**Benefits of kangaroo mother care**	
Makes baby warm and grow faster	10 (16.7)
Result in increased duration and rate of breastfeeding	5 (8.3)
Improves bonding between mother and baby	5 (8.3)
All of the above	40 (66.7)

The attitude of mothers towards the use of KMC was generally positive. Table 3 shows that during KMC, thirty-nine (65%) of the mothers felt their babies were safe during KMC while 21 (35%) felt their babies were not safe. Also, 39 (65%) of the respondents did not find KMC tiring. Majority of the mothers (75%) had good perception demonstrated by their being happy practicing KMC and knew how to correctly position the baby, for KMC (Table 4). All the respondents practiced KMC while in the ward. Table 5 shows the association between the variables and the utilization of KMC. The knowledge of mothers regarding KMC significantly influenced its use.

**Table 3:** respondents attitude towards the use of kangaroo mother care

Attitude of mothers towards the use of kangaroo mother care (KMC)	Frequency (%) N = 60	Percentage
**During KMC, did you feel that the baby was safe?**		
Yes	39 (65)	65
No	21 (35)	35
I don’t know	-	-
**Did you find KMC to be tiring?**		
Yes	21 (35)	35
No	39 (65)	65
I don’t know	-	-
**During KMC, did you think the baby would receive enough warmth?**		
Yes	35 (58.3)	58.3
No	25 (41.7)	41.7
I don’t know	-	-

**Table 4:** perception and practice of mothers on the use of kangaroo mother care

Variable	Frequency (%) N = 60
**Responses on how to strap a baby for KMC**	
Strap to the back	6 (10)
In between the breast/on the chest	49 (81.7)
On the abdomen	5 (8.3)
**Responses on how long KMC can be practiced within 24 hours**	
8-12 hours	10 (16.7
13-24hours	10 (16.7)
1 - 2hrs	40 (66.6)
As long as possible	-
**Respondents perception when baby was in Kangaroo position**	
Felt happy as a mother	45 (75)
Was scared baby may get suffocated	4 (6.7)
Had no particular feeling	4(6.7)
Was anxious	7 (11/6)

**Table 5:** influence of knowledge, attitude and practice of mothers on the use of kangaroo mother care

Variable	N	Df	Mean	SD	X2-calc	Sig.
Influence of knowledge of mothers on the use of kangaroo mother care	60	4	2.65	1.191	11.833	0.019
Influence of attitude of mothers on the use of kangaroo mother care	60	4	2.37	1.073	22.333	0.000
Influence of practice on kangaroo mother care	60	4	2.85	1.505	10.167	0.038

## Discussion

The present study assessed the knowledge, attitude and practice of KMC among post-natal mothers of preterm LBW infants. Our findings showed that there is high awareness and knowledge of KMC among our preterm mothers (up to 80%) with positive attitudes towards the practice. This was in line with a similar study [13] in India which showed high knowledge and positive attitude towards KMC among mothers in a tertiary care centre. Also among public health workers, Nagai *et al*. [16] demonstrated good knowledge of KMC. This is in contrast to some other studies in India, that reported poor knowledge of KMC in respondents [17, 18]. In Ghana, Nguah *et al*. also reported poor knowledge of KMC in postnatal mothers [19]. The reason for the disparity in knowledge may be due to poor awareness creation in the communities studied and also among the healthcare providers [20].

Most of these studies were also conducted in the rural settings. Our study was conducted in an urban tertiary care center designated as a center of excellence for KMC by Federal Ministry of Health because of its dedication and training of NICU mothers on KMC [15]. We noted that the majority of the mothers declared their source of information on KMC to be from the hospital (60%). With adequate health awareness creation on KMC postnatal mothers can be motivated and able to utilize KMC. This was demonstrated in the present study with more than half of the respondents (65%) indicating that KMC was worthwhile and not a waste of time. All the mothers practiced kangaroo mother care while in the ward. This was in contrast to the studies by Mfuh *et al*. in Northern Nigeria and Roba *et al*. in Eastern Ethiopia that showed 57% and 54% of respondents respectively practiced KMC in their facilities [18, 21]. This disparity may be due to the differences in knowledge of KMC by the participating mothers as poor knowledge may be a hindrance to KMC [13].

Majority of the respondents felt happy when their infants were in the kangaroo position. The study by Umila *et al*. in North Kerala, India also showed that more than 90% of respondents were happy and willing to provide KMC always [22]. The KMC position promotes bonding, stimulates the mother´s breast milk production and also brings the milk source closer and more accessible to the infant [7]. When mothers recognize that they are contributing to the survival of their preterm infants by providing warmth and preventing cold injuries to their infants, they get motivated. Adequate counselling and education on KMC is very important for uptake and sustained acceptance. In the present study, good knowledge of KMC by the mothers had a positive influence on the attitude and practice of KMC. This had been corroborated by other researchers elsewhere [13, 21]. If a mother understands the benefit of KMC to her baby she is more likely to be predisposed to offer that care to her baby. Kangaroo care seeks to provide and improve bonding between the newborn and the family members too as any of the family members can also provide KMC if mother is not available. This ensures both physiological and psychological warmth and bonding. All these factors have a profound impact on the baby´s well-being and survival. Thus KMC if properly utilized can help to reduce preterm complications, increase maternal satisfaction and ultimately reduce under 5 mortalities in low resource countries.

## Conclusion

The knowledge of KMC among mothers whose babies were admitted in the newborn wards of LUTH was high, they believed that KMC was helpful to their babies and were happy practicing it. It is recommended that more advocacy, education and training on KMC for post-natal mothers at all levels of health facilities should be encouraged and intensified for the benefit of the small babies and to reduce neonatal mortalities.

### What is known about this topic

- Kangaroo mother care promotes breastfeeding and can prevent preterm death;
- It is an effective and low-cost technique which prevents neonate from hypothermia, and promote child growth.

### What this study adds

- Kangaroo mother care improves the confidence of the mother to care for the preterm infant;
- Knowledge of kangaroo mother care has a positive influence on the attitude and practice of KMC.
